# Effect of Microalgal Extracts from *Chlorella vulgaris* and *Scenedesmus quadricauda* on Germination of *Beta vulgaris* Seeds

**DOI:** 10.3390/plants9060675

**Published:** 2020-05-26

**Authors:** Ivana Puglisi, Valeria Barone, Ferdinando Fragalà, Piergiorgio Stevanato, Andrea Baglieri, Alessandro Vitale

**Affiliations:** 1 Department of Agriculture, Food and Environment, University of Catania, 95123 Catania, Italy; ipuglisi@unict.it (I.P.); ferdinandofragala@virgilio.it (F.F.); alevital@unict.it (A.V.); 2 Department of Agronomy, Food, Natural Resources, Animals and Environment, University of Padova, Legnaro, 35020 Padova, Italy; valeriabarone89@alice.it (V.B.); stevanato@unipd.it (P.S.)

**Keywords:** microalgae, germination percentage, mean daily germination, seedling vigor index, total root length, priming treatment

## Abstract

Sugar beet (*Beta vulgaris* subsp. *vulgaris*) is a commercially important biennial root crop, providing about 20% of the world’s annual sugar production. Seed quality is crucial for adequate plant growth and production. The productivity of sugar beet is often limited by heterogeneous germination in the field. In order to improve the sugar beet germination process, the effect of different concentrations of microalgal extracts from *Chlorella vulgaris* or *Scenedesmus quadricauda* was investigated by calculating several indices useful to evaluate the germination performance. Moreover, root morphological analysis was performed by using WinRHIZO software. *B. vulgaris* seeds were soaked with five different concentrations (from 0.1 to 10 mg C_org_/L) of the microalgal extracts, considering the amount of organic carbon (C_org_) in each extract. Our results show that these microalgal extracts exert a positive effect on sugar beet germination, by increasing efficiency and regularity of this critical process for *B. vulgaris* seeds. The best results, in terms of germination indices as well as root morphological traits, were reached by using *C. vulgaris* extract at the concentrations C2 (1 mg C_org_/L) and C3 (2 mg C_org_/L).

## 1. Introduction

Seed germination is a crucial process, characterized by a series of steps, normally occurring before radicle emergence from the seed coat [1,2]. Radicle emergence percentage can deeply affect the production and the crop quality since the germination process is a sensitive step for plant growth and it is strictly linked to the seed quality, being the radicle emergence percentage related to seed germination potential [3,4,5]. In fact, during the germination of seeds, different enzymes such as amylases, proteases, and lipases lead to the hydrolysis of reserve substances, producing compounds that are transported to the growing seedlings for their development [6]. 

Sugar beet (*Beta vulgaris* subsp. *vulgaris*) is mainly grown in temperate climates and it is an important industrial biennial root crop, providing about 20% of the world’s annual sugar production and representing a source material for bioethanol and animal feed production [7]. Sugar beet seed health and quality are crucial for adequate plant growth and they are strictly associated with the productive yield both in terms of quantity and quality of the crop [8]. Unfortunately, the productivity of sugar beet is often limited by heterogeneous germination in the field, probably due to the presence of inhibitory substances in the pericarp of the seed as well as pathogen attacks [9,10]. Moreover, about 35–40% of the sugar beet seeds need to be discarded before sowing in the field because they are defective [11]. Finally, after germination, sugar beet seedlings often have to confront different biotic and abiotic stresses, therefore, rapid germination of sugar beet seeds is crucial for plant development and overall yield [12,13]. For this purpose, different techniques have been adopted in order to increase the physiological potential of sugar beet seeds and their treatment may become an indispensable procedure aiming at the enhancement of the seed vigor and the reduction of the variability in the germination process. Among these, seed priming with water, salicylic acid, or gibberellic acid has been shown to promote sugar beet seed germination as well as enhance sugar beet seedling growth [10]. Szajsner et al. [14] showed that the seed vigor, germination speed, and the germination time significantly improved by using a magnetic field or a semiconductor laser radiation as pre-sowing treatments of sugar beet seeds.

An aqueous extract from microalga *Acutodesmus dimorphus* was successfully used as a seed primer, improving the germination energy of tomato seeds (cv. Roma) and increasing lateral root development and extract concentrations [15]. Conversely, other authors found that higher seaweed extract concentrations have caused inhibition of seed germination [16,17,18]. 

Recently, Barone et al. [19] have shown that extracts from microalgae *Chlorella vulgaris* or *Scenedesmus quadricauda* may act as biostimulant in the early stages of sugar beet cultivation, when added after 5 days to the Hoagland growth solution, by improving root and plant growth and modulating gene expression related to the nutrient acquisition in sugar beet. Moreover, these extracts were successfully applied at the root level in the cultivation substrates, showing to exert a biostimulant effect on tomato and lettuce seedlings [20,21]. 

Considering that *C. vulgaris* and *S. quadricauda* extract have been successfully applied as a biostimulant to several crops, including sugar beet, the aim of this work was to evaluate whether these microalgae extracts may also be able to positively affect the germination process as a priming treatment for sugar beet seed. Moreover, the concentration-dependent effects of microalgal extracts from *C. vulgaris* or *S. quadricauda* were also evaluated. Therefore, seed germination of *B. vulgaris* was monitored with the aim to calculate several physiological indices, useful to evaluate the effect of pre-soaking seeds with the microalgal extracts. In addition, root morphological traits were also evaluated by using WinRHIZO software in order to evaluate the effectiveness of these priming treatments. 

## 2. Results and Discussions

Several germination indices of sugar beet seeds were calculated and monitored (as detailed in the Materials and Methods section) in order to evaluate the effect of the treatments and their concentration dependence, using five amounts of *C. vulgaris* (CVextr) or *S. quadricauda* extracts (SQextr). 

In all data analysis, a significant effect (*p* < 0.01) of microalgal extract concentration was observed on all germination variables of *B. vulgaris* seedlings. Similarly, all concentration × microalgal extract interactions were significant (*p* < 0.01) for all germination parametric indices. Therefore, the experiments were always presented for each type of microalgal extract (*C. vulgaris* or *S. quadricauda*).

**Figure 1:**
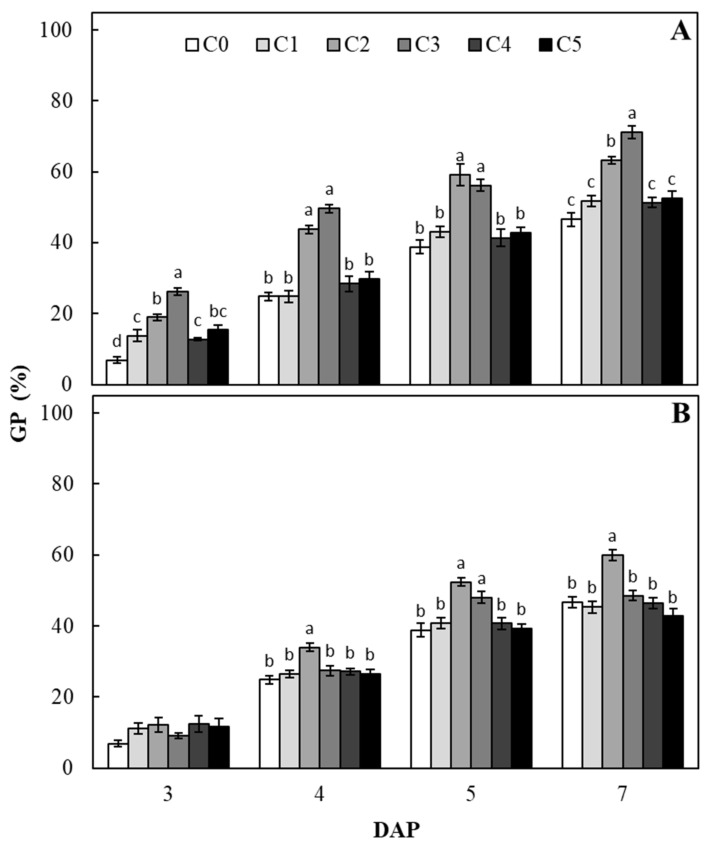
Germination percentage (GP) of sugar beet seeds treated with different concentrations of *C. vulgaris* (**A**) or *S. quadricauda* (**B**) extracts, for each day after priming (DAP). C0 = 0 mg C_org_/L; C1= 0.1 mg C_org_/L; C2 = 1 mg C_org_/L; C3 = 2 mg C_org_/L; C4 = 5 mg C_org_/L; C5 = 10 mg C_org_/L. Data (± standard error bar) are the means of three replicates (each formed by 100 seeds). Columns within each sampling point followed by the same letters are not significantly different according to Fisher’s least significant difference test (α = 0.05). The absence of letters above the columns shows the lack of significant differences.

The percentage of sugar beet seed germination (GP) is reported in Figure 1. All the *C. vulgaris* extract-based treatments significantly affected the GP values in comparison to the untreated seeds (C0), 3 days after priming (DAP) (Figure 1A). In particular, C2 and C3 *C. vulgaris* extract concentrations increased GP values, showing at 3 DAP a significant increase with respect to the control of 2.8 and 3.8 folds, respectively. At the remaining monitoring times, only C2 and C3 *C. vulgaris* extract concentrations significantly affected GP values in comparison to the untreated seeds, reaching the highest values at 4 DAP, when GP values calculated for C2 and C3 were 1.8 and 2 times higher than those calculated in the control seeds, respectively. The final GP values, calculated 7 DAP, were 1.4 (C2) and 1.5 (C3) times higher than those calculated in the untreated seeds. Conversely, among the different *S. quadricauda* extract concentrations, the effectiveness of the treatment was observed only after 4 DAP (Figure 1B). From 4 to 7 DAP, C2 *S. quadricauda* extract concentration positively affected seed germination, by increasing the GP values of around 1.3 times with respect to the untreated seeds. Noteworthy also that C3 concentration significantly increased GP value with respect to the untreated seeds (1.2 folds), limited to 5 DAP (Figure 1B). 

**Figure 2:**
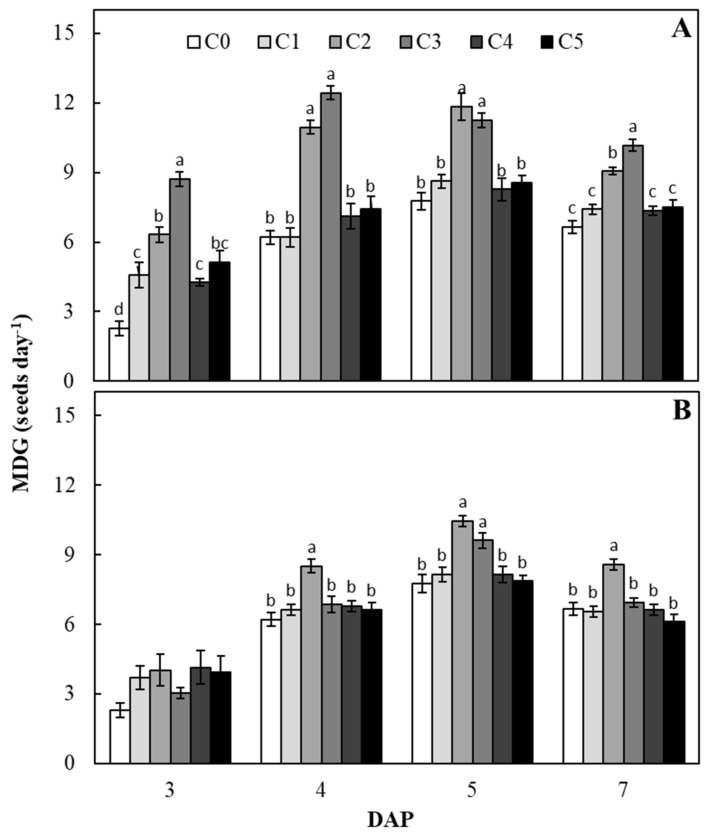
Mean daily germination (MDG) of sugar beet seeds treated with different concentrations of *C. vulgaris* (**A**) and *S. quadricauda* (**B**) extracts, for each day after priming (DAP). C0 = 0 mg C_org_/L; C1 = 0.1 mg C_org_/L; C2 = 1 mg C_org_/L; C3 = 2 mg C_org_/L; C4 = 5 mg C_org_/L; C5 = 10 mg C_org_/L. Data (± standard error bar) are the means of three replicates (each formed by 100 seeds). Columns within each sampling point followed by the same letters are not significantly different according to Fisher’s least significant difference test (α = 0.05). The absence of letters above the columns shows the lack of significant differences.

Results reported in Figure 2 show that at all the monitoring times, mean daily germination (MDG) values calculated for seeds treated with C2 and C3 *C. vulgaris* extract concentrations, were always greater than those calculated for untreated seeds, reaching the highest increase only after 3 DAP (2.8 and 3.8 times higher than those calculated in control seeds for C2 and C3, respectively) (Figure 2A). Among *S. quadricauda* extract concentrations, at all monitoring times except for 3 DAP, the highest MDG values, if compared to that calculated in untreated seeds, were recorded in seeds treated with C2, showing always an increase of 1.3 times (Figure 2B).

These results suggest that treatments exerted different effects on GP and MDG values, strictly related to the microalgae species as well as the extract concentrations. Interestingly, the positive effect on GP values, mainly observed by using C2 and C3 concentrations of CVextr and C2 concentration of SQextr, were higher than those obtained by Szajsner et al. [14], who found GP values around 1 fold higher than controls by treating sugar beet seeds with a magnetic field or laser radiation. Therefore, these results indicate that these priming treatments, in particular C2 and C3 CVextr, may be a promising alternative practice aiming to enhance seed germination performance and increase GP and MDG indices, strictly related to the variability of this important physiological process. Interestingly, the lower and higher extract concentrations (C1, C4, and C5) do not significantly affect the GP and MDG germination indices, as these values were always similar to the respective controls. These data are in accordance with that reported by Santos et al. [22], who found that the application of algae *Ascophyllum nodosum* extract-based biostimulants in ornamental sunflower requires an optimal concentration to increase germination indices. Conversely, an aqueous extract from microalga *Acutodesmus dimorphus* behaved as an effective primer on tomato seeds, at increasing extract concentrations [15]. Indeed, natural biostimulants may contain various biologically active compounds which may determine concentration-dependent effects, making crucial the testing of a broad range of concentrations [23]. Moreover, depending on the extract type and concentration applied, natural biostimulants may elicit different responses in treated plants, being sometimes also potentially phytotoxic [24]. Our data suggest that the microalgal extracts did not negatively affect the germination process at lower and higher concentrations, showing no phytotoxic effect, as supported by Ronga et al. [25], who found no phytotoxicity in an aqueous *C. vulgaris* microalgal extract on cress. 

Interestingly, the two extracts showed an evident divergent effect on germination indices probably due to the difference in extract compositions [19]. Indeed, the two extracts showed a different composition in terms of organic carbon distribution (Supplementary Table S1) and element composition (Supplementary Table S2), as reported in Barone et al. [19]. In particular, the degree of hydrophobicity for humic substances determined according to Baglieri et al. [26], resulted in being much higher for CVextr (6.1) than that calculated for SQextr (3.8), showing CVextr to be more apolar than SQextr (Supplementary Table S1). These data suggest that the different extract compositions in organic carbon distribution as well as polarity, may be strictly related to the different effect on GP and MDG germination indices, as confirmed by Piccolo et al. [27], who found a relationship between the structure of the formulate and its bioactivity in humic substances.

**Figure 3:**
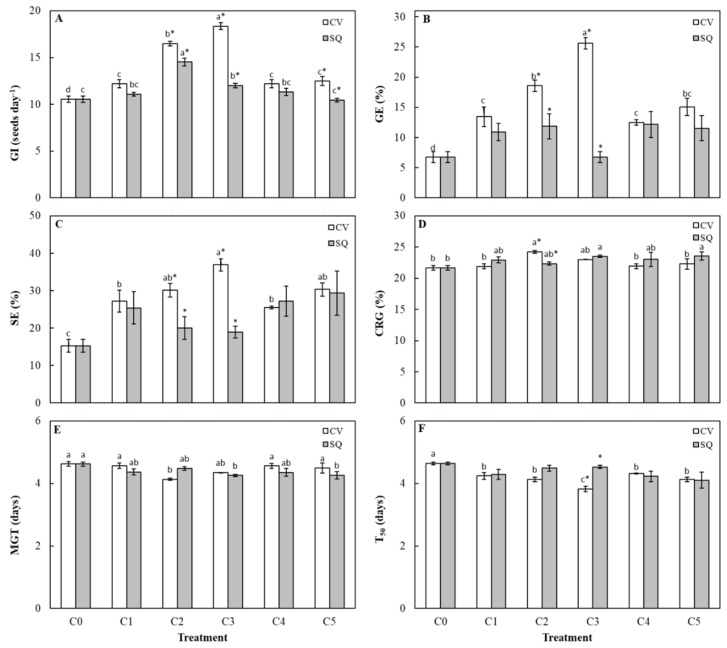
Germination index (GI) (**A**), germination energy (GE) (**B**), speed of emergence (SE) (**C**), coefficient of the rate of germination (CRG) (**D**), mean germination time (MGT) (**E**) and time required for 50% germination (T_50_) (**F**) of sugar beet seeds treated with different concentrations of *C. vulgaris* (CV) and *S. quadricauda* (SQ) extracts. C0 = 0 mg C_org_/L; C1 = 0.1 mg C_org_/L; C2 = 1 mg C_org_/L; C3 = 2 mg C_org_/L; C4 = 5 mg C_org_/L; C5 = 10 mg C_org_/L. Data (± standard error bar) are the means of three replicates. The same colored columns representing 5 concentrations of each algal extract followed by the same letters are not significantly different according to Fisher’s least significant difference test (α = 0.05). The absence of letters above the columns shows the lack of significant differences. The presence of an asterisk (*) within each concentration show a significant difference between the two algal extracts.

Data reported in Figure 3 show that the two concentrations C3 and C2 of *C. vulgaris* extract significantly increased the germination indices (GI) (1.7 and 1.6 times, respectively), germination energy (GE) (3.8 and 2.8 times, respectively), speed of emergence (SE) (2.4 and 2 times, respectively) and coefficient of the rate of germination (CRG) (around 1.1 times for both concentrations) (Figure 3A, B, C and D), if compared to the control; on the other hand, mean germination time (MGT) and T_50_ (Figure 3E and F) were significantly reduced (around 1.1 times for both indices and concentrations). As regards to the T_50_ index, all the *C. vulgaris* extract concentrations significantly reduced the time required for 50% germination (Figure 3F). These findings support the hypothesis that the treatment with *C. vulgaris* extract at all the concentrations tested may be a very useful priming treatment in order to improve seed germination performance, in terms of reduction of the time required to obtain 50% seed germination. Indeed, it is well known that the higher the GI, GE, SE, and CRG values, the higher the positive effect on seed germination [28]. In contrast, the lower the T_50_ and MGT values with respect to the control, the lower the inhibition on seed germination [28]. As regards the *S. quadricauda* extract, the concentration effects on germination indices were less evident and differences among concentrations were not always significant (Figure 3). Nevertheless, the concentration C2 positively affected GI index (1.4 times) with respect to the control, and the CRG index values were significantly higher in seeds treated with C3 and C5 concentrations than those calculated for untreated seeds. Conversely, MGT values were significantly reduced by the treatments with C3 and C5 concentrations (around 1.1 for both amounts of extract). All other germination indices were not significantly affected by *S. quadricauda* extract at all the tested concentrations (Figure 3). These data show that *S. quadricauda* extract also positively affected seed germination, although to a lesser extent than *C. vulgaris* extract. In detail, the calculated GI, GE, and SE values were almost always lower than those relative to the corresponding concentrations of *C. vulgaris*, moreover, T_50_ values were not positively affected by *S. quadricauda* extract, showing values always similar to those calculated for the control (Figure 3F). It is noteworthy to underline that these differences between the two algal extracts were significant only for the C2, C3, and C4 concentrations with regard to GI, for the C2 and C3 concentrations with regard to GE and SE indices, for C2 concentration with regard to CRG and MGT, and for C3 concentration with regard to the T_50_ index (Figure 3). These results show that CVextr seems to be more effective than SQextr, with C2 and C3 the optimal concentrations, although a higher amount of CVextr did not negatively affect seed germination. These findings are supported by Ronga et al [25], who found that a two-fold concentration (around 25 mgC_org_/L) of an aqueous *C. vulgaris* microalgal extract did not show phytotoxicity effect, by measuring the GI index, on a sensitive species to phytotoxic compounds such as cress. Interestingly, from a physiological point of view, several authors [29,30,31,32] have reported that the faster emergence in sugar beet showed an enormous influence on plant characteristics, showing a higher dry matter weight compared to the plants emerging later. Among these authors, Podlaski et al. [32] demonstrated that the time of emergence was the strongest factor influencing plant weight in sugar beet during harvest season.

Analysis of morphological data provided always a significant effect (*p* < 0.01) of microalgal extract concentrations on all morpho-biometric parameters (length, surface area, mean root diameter, root volume, tips, root 0.000 < L < 0.500 and root 0.500 < L < 1.000) of *B. vulgaris* seedlings. Since concentration × microalgal extract interactions were significant for all parametric variables, the data were presented for each microalgal extract (Table 1 and Table 2). 

The positive effect of C2 and C3 *C. vulgaris* extract concentrations were also confirmed by morphological parameters (Table 1). In particular, all *C. vulgaris* extract concentrations significantly increased all root morphological parameters, both after 5 and 7 DAP, being the C2 and C3 CVextr the highest values, in comparison to the control (Table 1). The positive effect of C2 and C3 *C. vulgaris* extract concentrations were also observed at the root volume level, whereas mean root diameter values were unaffected or reduced by the treatments (Table 1). Similarly, all root morphological traits were positively affected by *S. quadricauda* extracts too (Table 2), although to a lesser extent than *C. vulgaris*. Noteworthy, the highest values of morpho-biometric parameters were reached using C2 and C3 *S. quadricauda* extract concentrations, whereas each amount of the SQextr did not positively affect the mean root diameter (Table 2), as it was already observed using *C. vulgaris* extract (Table 1). These results are in accordance with Barone et al. [19], who found that by applying to the hydroponic solution 1 and 2 mg Corg/L of the two microalgal extracts (*C. vulgaris* or *S. quadricauda*), root apparatus of sugar beet seedlings was positively affected by increasing total root length, root surface area, and the number of root tips, whereas the average diameter and the volume of roots were not affected by the treatments. The effect on root morphology may be related to the degree of hydrophobicity of extracts (Supplementary Table S1), the latter being closely related to the increase of root growth [27]. *C. vulgaris* extract showed a degree of hydrophobicity value (6.1) greater than those observed for humic substances of a different origin, ranging between 0.61 and 4.75 [33], whereas *S. quadricauda* extract showed an intermediate value (3.8). In particular, methoxilic groups, aryl groups, and carboxylic acids seem to be involved in the bioactivity of natural biostimulant substances, and often related to hormone-like compounds [27]. Therefore, both extracts, on the basis of their characterization, seem to be perfectly compatible with the effect observed on the morphological traits in sugar beet roots [34]. Moreover, the performances obtained in the present study seem to be higher than those obtained by Szajsner et al. [14], who treated sugar beet seeds with a magnetic field or laser radiation. In particular, Szajsner et al. [14] achieved an increase of the seedling length of 1.5 times with respect to the control after 4 days from the pre-sowing treatments, whereas the treatment with C3 *C. vulgaris* extract, induced an increase of the seedling length of around 6 times with respect to the untreated seeds at 5 days after priming treatment (Table 1). 

**Figure 4:**
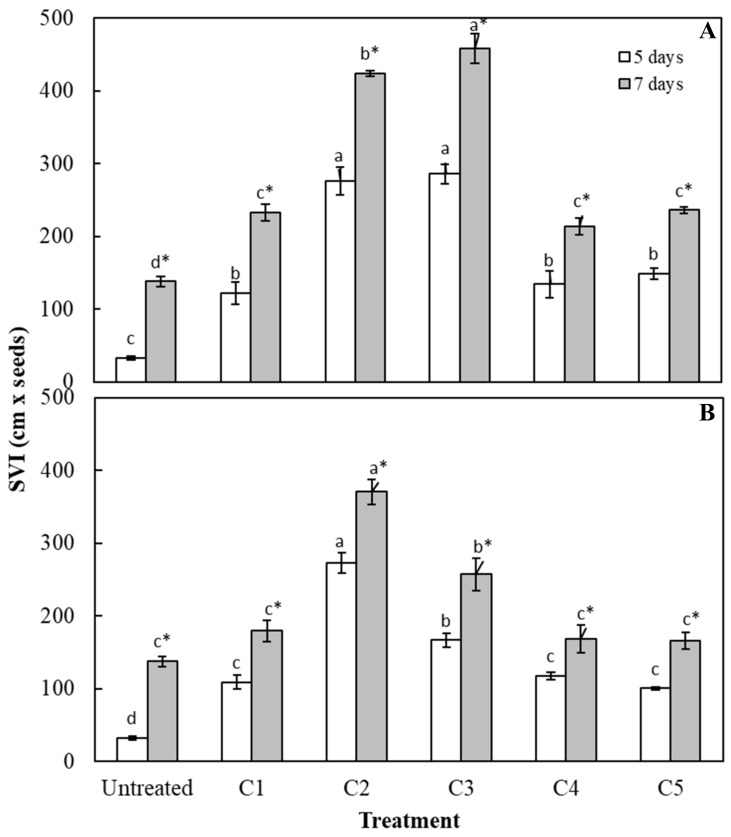
Seedling vigor index (SVI) after 5 and 7 days of sugar beet treated with different concentrations of *C. vulgaris* (**A**) and *S. quadricauda* (**B**) extracts. C0 = 0 mg C_org_/L; C1 = 0.1 mg C_org_/L; C2 = 1 mg C_org_/L; C3 = 2 mg C_org_/L; C4 = 5 mg C_org_/L; C5 = 10 mg C_org_/L. Data (± standard error bar) are the means of three replicates. The same colored columns representing 5 concentrations of each algal extract followed by the same letters are not significantly different according to Fisher’s least significant difference test (α = 0.05). The presence of an asterisk (*) within each concentration indicates a significant difference between the two algal extracts.

Finally, SVI values were also calculated both at 5 and 7 days after priming treatments (Figure 4). All CVextr treatments positively affected SVI values, in accordance with other calculated germination indices. In particular, the C3 *C. vulgaris* extract resulted in being the most performant treatment, determining an increase of seedling vigor index (SVI) of around 9 and 3 times higher than the controls after 5 and 7 days, respectively. As regards to *S. quadricauda* extract, C2 induced the greatest increase of 8 and 3 times higher than the controls after 5 and 7 days, respectively (Figure 4). These results suggest that the use of these microalgae extract as priming treatment, may be a good alternative to other priming methods adopted for sugar beet, according to Islam et al. [28], who observed that the higher the seedling vigor index (SVI) value, the higher the positive effect on seed germination. Moreover, in accordance with Ugena et al. [35], pre-sowing treatment with different biostimulant compounds, aiming to increase the vigor of seedlings, represents an innovative alternative to cope with different kinds of stresses. Comprehensively, all these results taken together suggest that best results, in terms of the germination process as well as root morphological traits, were reached by using the concentrations C2 and C3 of *C. vulgaris* extract, showing that this microalgal extract, besides exerting a biostimulant effect when added to the growth medium of sugar beet seedlings [19], may also be used as a priming method positively affecting the sugar beet seed germination.

## 3. Materials and Methods

### 3.1. Microalgae Cultivation and Extract Preparation

The microalgae used in this study were *C. vulgaris* (Beijerinck, CCAP 211/11C) and *S. quadricauda* (isolated from an algal company raceway pond, located in Borculo, Gelderland, the Netherlands in 2011). They were obtained by and maintained in the algal collection of the Department of Agriculture, Food and Environment (Di3A) (University of Catania, Italy) as described in Baglieri et al. [36]. Microalgal growth was conducted in 250 mL flask containing 150 mL of sterile BG11 culture medium [37] at pH 8.4, incubated on a mechanical shaker (100 rpm) at 25–30 °C, illuminated by a 3500-lx, average photon flux (PPF) 100 μmol photons m^−2^ s^−1^ light source (PHILIPS SON-T AGRO 400) with a 12 h photoperiod for 30 days in a growth chamber and aerated by pumps with 20 L h^–1^ 1.5% CO_2_ [38]. Microalgal biomasses were harvested by centrifugation (at 5000 rpm for 15 min), washed with distilled water (up conductivity < 200 μS cm^−1^), and freeze-dried as described in Puglisi et al. [39]. 

Microalgal extract stock solutions (referred to as CVextr and SQextr) were prepared as described in Barone et al. [19]. Briefly, microalgae cells were collected and centrifuged at 5000 rpm for 15 min and the final pellets obtained from each microalgal biomass were added to methanol to lyse the cell wall in order to obtain the intracellular extracts. After centrifugation and evaporation of the organic solvent, the extracts were freeze-dried and collected with distilled water to obtain the microalgal extract stock solution. The complete characterization of extracts was reported in Barone et al. [19]. 

### 3.2. Plant Material and Experimental Conditions 

The sugar beet variety used in this study is the hybrid “Shannon” provided by the Department of Agronomy, Food, Natural Resources, Animals and Environment (DAFNAE) of the University of Padova (Italy) [40]. Seeds were soaked in 76% ethanol for 5 min, rinsed with sterilized water, and placed on distilled water moistened filter paper. The treatments were performed by diluting different amounts of microalgal extracts in distilled water, used to moisten the filter paper. For each microalgal extract, five different concentrations were tested and they were calculated on the basis of the extract organic carbon (C_org_) content: C1 = 0,1 mg C_org_/L; C2 = 1 mg C_org_/L; C3 = 2 mg C_org_/L; C4 = 5 mg C_org_/L; C5 = 10 mg C_org_/L. Control samples using untreated seeds (C0 = 0 mg C_org_/L) were routinely performed. Germination was carried out in a growth chamber in the dark at 25 °C. Sugar beet seeds were considered germinated when a radicle of at least 2 mm emerged. The germinated seeds were counted and monitored daily for 7 days, after this time no germination, even of those not yet germinated, was detected. The experimental procedure was repeated twice in a complete randomized block design and for each treatment, four replicates consisting of 100 seeds were tested according to the methods of the International Rules for Seed Testing [41,42].

### 3.3. Germination Indices

In order to evaluate the effect of microalgal extract treatments, several germination indices were calculated as detailed below.

The germination percentage (GP) was calculated for each treatment as a percentage of total germinated seeds after 3, 4, 5, and 7 days after priming (DAP):

(1) $$\mathrm{GP}=\left(\mathrm{number}\;\mathrm{of}\;\mathrm{germinated}\;\mathrm{seeds}/\mathrm{number}\;\mathrm{of}\;\mathrm{total}\;\mathrm{seeds}\;\mathrm{for}\;\mathrm{bioassay}\right)\times 100$$

The mean daily germination (MDG), representing the mean number of seeds germinated per day, was calculated at 3, 4, 5, and 7 DAP [43]:

(2) MDG=GP/t

where GP is the germination percentage, and t is the number of DAP.

The germination index (GI), also known as mean germination rate or rate of Maguire [44], is a measure assigning the maximum arithmetic weight to seeds that germinate at the first days of count and less weight to those germinating later. GI was calculated as follows:

(3) $$\mathrm{GI}=\left[\mathrm{number}\;\mathrm{of}\;\mathrm{germinated}\;\mathrm{seeds}/\mathrm{days}\;\mathrm{of}\;\mathrm{first}\;\mathrm{count}\right]+\ldots \;+\left[\mathrm{number}\;\mathrm{of}\;\mathrm{germinated}\;\mathrm{seeds}/\mathrm{days}\;\mathrm{of}\;\mathrm{final}\;\mathrm{count}\right]$$

The mean germination time (MGT) was calculated according to Soltani et al. [45] as follows:

(4) $$\mathrm{MGT}=\sum \left(n,t\right)/\sum n$$

where n is the number of newly germinated seeds at time t.

The germination energy (GE) was calculated according to Ruan et al. [46] as follows:

GE = (Percentage of germinated seeds at the starting day of germination / Total number of seeds sets for bioassay) × 100.(5)

The speed of emergence (SE), was calculated according to Islam et al. [28] as follows:

SE = (Number of germinated seeds at the starting day of germination / Number of germinated seeds at the final days of measurement) × 100.(6)

The coefficient of the rate of germination (CRG) was calculated according to Chiapusio et al. [47] as follows:

(7) $$\mathrm{CRG}=\left[\left(N_{1}+N_{2}+\ldots +N_{n}\right)/\left(N_{1}\times T_{1}\right)+\left(N_{2}\times T_{2}\right)+\ldots +\left(N_{n}\times T_{n}\right)\right]\times 100$$

where N_1_, N_2_, …, N_n_ are the number of germinated seeds on time T_1_, T_2_, …, T_n_.

The time required for 50% germination (T_50_), was calculated according to Coolbear et al. [48] as follows:

(8) $$T_{50}=t_{i}+\left[\left(\left(N/2\right)-n_{i}\right)\times \left(t_{i}-t_{j}\right)\right]/(n_{i}-n_{j})$$

where N is the final number of germinated seeds, n_i_ and n_j_ the cumulative numbers of seeds germinated by adjacent counts at times t_i_ and t_j_. 

Finally, the seedling vigor index (SVI) was calculated at 5 and 7 DAP, according to Noorhossein et al. [43] as follows:

SVI = GP × seedling length.(9)

### 3.4. Root Morphological Analysis

Root morphological analysis was performed by using WinRHIZO software (Regent Instruments) and total root length, root surface area, mean root diameter, root volume, total number of root tips, lateral root (0.000 < L < 0.500 and 0.500 < L < 1.000) were determined by computerized scanning (STD 1600, Regent Instruments, Canada) at 5 and 7 days from sowing [49].

### 3.5. Statistical Analysis

Data on performances of microalgal extracts from the repeated experiment were analyzed by using the Statistica package software (version 10; Statsoft Inc., Tulsa, OK, USA). The arithmetic means of parameters were calculated, by averaging the values determined for the single replicates of each treatment. Percentage data were transformed into the arcsine (sin^−1^ square root x) prior to the analysis of variance (ANOVA). Initial analyses were performed by calculating associated F and P values to evaluate whether the effects of a single factor (concentration) and microalga × concentration interactions were significant. In the post-hoc analysis, the corresponding mean values of all parameters were subsequently separated by Fisher’s least significant difference test (*p* = 0.05). Untransformed arithmetic means of parameters are presented in the figures and tables.

## 4. Conclusions

This study leads us to employ microalgae extracts as a priming agent in order to improve the germination process of an important industrial crop such as sugar beet. The novelty of this work consists of the successful application of *C. vulgaris* and *S. quadricauda* as pre-sowing treatments, resulting in the first work in which microalgae extracts are employed as a priming method for sugar beet seed germination. Microalgae extracts, especially *C. vulgaris*, was found to improve all calculated germination indices, the root traits as well as the seedling vigor of sugar beet, putatively promoting the further nutrient acquisition and plant growth. Among the tested extract concentrations, 1 mg C_org_/L and 2 mg C_org_/L of *C. vulgaris* extract revealed to be the best priming treatments, exerting a positive effect both on the germination process and morphological traits of sugar beet seedlings. Although further investigations should be performed, based on our findings, *C. vulgaris* extract may represent a promising practice to increase the physiological potential of sugar beet seeds. 

## Figures and Tables

**Table 1:** Performance of different dosages of *Chlorella vulgaris* extract on different morpho-biometric parameters on *Beta vulgaris* seedlings. Data derived from two repeated experiments. Standard error of the means = SEM, *n* = 4. Means followed by different letters within the column are significantly different according to Fisher’s least significant difference test (α = 0.05).

	Length (cm)		Surface Area (cm^2^)		Mean Root Diameter (mm)		Root Volume (cm^3^)		Tips Number		Root Number (0.000 < L < 0.500)		Root Number (0.500 < L < 1.000)	
	5 days	7 days	5 days	7 days	5 days	7 days	5 days	7 days	5 days	7 days	5 days	7 days	5 days	7 days
**C0**	0.8 ± 0.1 d	3.0 ± 0.2 c	0.2 ± 0.01 e	0.3 ± 0.01 c	0.6 ± 0.01 ab	0.6 ± 0.02 a	0.003 ± 0.0 d	0.01 ± 0.0 ab	1.5 ± 0.07 e	2.9 ± 0.1 e	0.2 ± 0.04 b	1.4 ± 0.1 d	0.6 ± 0.02 c	1.4 ± 0.1 a
**C1**	2.8 ± 0.2 c	4.5 ± 0.2 b	0.5 ± 0.05 d	0.6 ± 0.05 b	0.6 ± 0.02 b	0.4 ± 0.04 bc	0.01 ± 0.0 c	0.01 ± 0.0 bc	3.0 ± 0.14 d	5.04 ± 0.2 c	3.0 ± 1.1 a	3.2 ± 0.2 bc	1.2 ± 0.2 b	1.2 ± 0.2 a–c
**C2**	4.7 ± 0.1 a	6.7 ± 0.1 a	0.8 ± 0.01 b	0.9 ± 0.03 a	0.6 ± 0.01 b	0.4 ± 0.02 c	0.01 ± 0.0 ab	0.01 ± 0.0 a	3.7 ± 0.22 c	8.0 ± 0.2 b	2.3 ± 0.6 a	5.3 ± 0.04 a	2.1 ± 0.2 a	1.3 ± 0.06 a–c
**C3**	5.1 ± 0.1 a	6.4 ± 0.2 a	0.9 ± 0.02 a	0.9 ± 0.01 a	0.6 ± 0.02 b	0.4 ± 0.01 c	0.01 ± 0.0 a	0.01 ± 0.0 a–c	4.6 ± 0.18 a	11.2 ± 0.2 a	2.7 ± 0.4 a	5.3 ± 0.2 a	2.3 ± 0.2 a	1.05 ± 0.1 bc
**C4**	3.2 ± 0.2 bc	4.2 ± 0.1 b	0.6 ± 0.02 c	0.6 ± 0.01 b	0.6 ± 0.02 a	0.5 ± 0.01 b	0.01 ± 0.0 b	0.01 ± 0.0 a–c	4.3 ± 0.14 ab	4.4 ± 0.1 d	1.7 ± 0.3 ab	2.7 ± 0.2 c	1.2 ± 0.1 b	1.3 ± 0.1 ab
**C5**	3.5 ± 0.1 b	4.5 ± 0.2 b	0.6 ± 0.02 c	0.6 ± 0.01 b	0.6 ± 0.02 ab	0.4 ± 0.01 c	0.01 ± 0.0 b	0.01 ± 0.0 c	4.1 ± 0.11 bc	5.2 ± 0.2 c	2.5 ± 0.7 a	3.4 ± 0.2 b	1.2 ± 0.1 b	1.0 ± 0.1 c

**Table 2:** Performance of different dosages of *Scenedesmus quadricauda* extract on different morpho-biometric parameters on *Beta vulgaris* seedlings. Data derived from two repeated experiments. Standard error of the means = SEM, *n* = 4. Means followed by different letters within the column are significantly different according to Fisher’s least significant difference test (α = 0.05).

	Length (cm)		Surface Area (cm^2^)		Mean Root Diameter (mm)		Root Volume (cm^3^)		Tips Number		Root Number (0.000 < L < 0.500)		Root Number (0.500 < L < 1.000)	
	5 days	7 days	5 days	7 days	5 days	7 days	5 days	7 days	5 days	7 days	5 days	7 days	5 days	7 days
**C0**	0.8 ± 0.1 d	3.0 ± 0.2 d	0.2 ± 0.01 c	0.3 ± 0.01 d	0.6 ± 0.01 b	0.6 ± 0.02 a	0.003 ± 0.0 c	0.01 ± 0.0 ab	1.5 ± 0.07 d	2.9 ± 0.1 b	0.2 ± 0.04 c	1.4 ± 0.1 e	0.6 ± 0.02c	1.4 ± 0.1 bc
**C1**	2.7 ± 0.1 c	3.9 ± 0.1 c	0.5 ± 0.01 b	0.6 ± 0.03 c	0.6 ± 0.01 b	0.5 ± 0.01 c	0.01 ± 0.0 ab	0.01 ± 0.0 b	2.5 ± 0.11 c	3.1 ± 0.1 b	1.1 ± 0.1 a	2.8 ± 0.1 bc	1.9 ± 0.1a	1.3 ± 0.1 c
**C2**	5.2 ± 0.3 a	6.2 ± 0.2 a	0.7 ± 0.03 a	0.9 ± 0.03 a	0.7 ± 0.01 ab	0.5 ± 0.01 c	0.01 ± 0.0 bc	0.01 ± 0.0 a	2.8 ± 0.13 c	5.3 ± 0.2 a	0.5 ± 0.2 bc	4.1 ± 0.2 a	1.2 ± 0.4b	1.9 ± 0.1 a
**C3**	3.5 ± 0.2 b	5.4 ± 0.3 b	0.7 ± 0.04 a	0.9 ± 0.01 a	0.6 ± 0.04 b	0.5 ± 0.02 c	0.01 ± 0.0 a	0.01 ± 0.0 a	3.4 ± 0.14 b	5.0 ± 0.2 a	1.2 ± 0.3 a	3.3 ± 0.2 b	2.1 ± 0.1a	1.8 ± 0.1 a
**C4**	2.9 ± 0.04 c	3.6 ± 0.2 c	0.5 ± 0.02 b	0.7 ± 0.02 b	0.7 ± 0.02 b	0.6 ± 0.04 ab	0.01 ± 0.0 a	0.01 ± 0.0 ab	4.0 ± 0.12 a	4.7 ± 0.2 a	1.0 ± 0.2 ab	1.9 ± 0.2 de	2.1 ± 0.1a	1.6 ± 0.04 a–c
**C5**	2.6 ± 0.1 c	3.9 ± 0.1 c	0.5 ± 0.01 b	0.7 ± 0.01 b	0.8 ± 0.06 a	0.5 ± 0.3 bc	0.01 ± 0.0 a	0.01 ± 0.0 ab	2.6 ± 0.3 c	5.1 ± 0.4 a	0.6 ± 0.1 bc	2.5 ± 0.2 cd	1.8 ± 0.1 a	1.8 ± 0.3 ab

## Supplementary Materials

The following are available online at https://www.mdpi.com/2223-7747/9/6/675/s1, Table S1: Distribution of C intensity of 13C NMR (%) of *Chlorella vulgaris* (CVextr) and *Scenedesmus quadricauda* (SQextr) extracts; Table S2: Element composition (%) of *Chlorella vulgaris* (CVextr) and *Scenedesmus quadricauda* (SQextr) extracts.

## References

[1] Mayer A.M., Shain Y. (1974). Control of seed germination. Ann. Rev. Plant Physiol..

[2] Martin R.C., Pluskota W.E., Nonogaki H. (2009). “Seed Germination” in Plant Developmental Biology—Biotechnological Perspectives.

[3] Abdolrahmani B., Ghasemi Golezani K., Valizadeh M., Feizi Asl V., Tavakoli A. (2011). Effect of seed priming on the growth trend and grain yield of barley (*Hordeum vulgare* L.) cv. Abidar under rainfed conditions. Seed Plant Prod. J..

[4] Najafi G., Khomari S., Javadi A. (2015). Germination response of canola seeds to seed vigor changes and hydro-priming. Seed Res..

[5] Rehman H., Iqbal H., Basra S.M.A., Afzal I., Farooq M., Wakeel A., Ning W. (2015). Seed priming improves early seedling vigor, growth and productivity of spring maize. J. Int. Agric..

[6] Millerd A., Thomson J. (1975). Storage proteins of legume seeds: Proteins of legume seeds: Potential for change. CSIRO Div. Plant Ind. Genet. Rep..

[7] Dohm J.C., Minoche A.E., Holtgräwe D., Capella-Gutiérrez S., Zakrzewski F., Tafer H., Rupp O., Sörensen T.R., Stracke R., Reinhardt R. (2014). The genome of the recently domesticated crop plant sugar beet (Beta vulgaris). Nature.

[8] Zahoor A., Faridullah S.P., Kakar K.M., Sanaullah B., El-Sharkawi H., Honna T., Yamamoto S. (2007). Sugar beet (*Beta vulgaris* L.) response to different planting methods and row geometries I. Effect on plant growth and yield. Arch. Agron. Soil Sci..

[9] Vanni A., Anfossi L., Cignetti A., Baglieri A., Gennari M. (2006). Degradation of pyrimethanil in soil: Influence of light, oxygen, and microbial activity. J. Environ. Sci. Health B.

[10] Dotto L., Silva V.N. (2017). Beet seed priming with growth regulators. Semina: Ciências Agrárias.

[11] Kockelmann A., Meyer U., Draycott A.P. (2006). Seed production and quality. Sugar Beet.

[12] Biancardi E., McGrath J.M., Panella L.W., Lewellen R.T., Stevanato P., Bradshaw J. (2010). Sugar Beet. Handbook of Plant Breeding, Tuberand Root Crops.

[13] Norouzi P., Stevanato P., Mahmoudi S.B., Fasahat P., Biancardi E. (2017). Molecular progress in sugar beet breeding for resistance to biotic stresses in sub-arid conditions-current status and perspectives. J. Crop Sci. Biotech..

[14] Szajsner H., Prośba-Białczyk U., Sacała E., Koszelnik-Leszek A., Szubzda B. (2017). The effect of pre-sowing seed stimulation on the germination and pigment content in sugar beet (*Beta vulgaris* L.) seedlings leaves. Pol. J. Natur. Sci..

[15] Garcia-Gonzalez J., Sommerfeld M. (2016). Biofertilizer and biostimulant properties of the microalga *Acutodesmus dimorphus*. J. Appl. Phycol..

[16] Kumar G., Sahoo D. (2011). Effect of seaweed liquid extract on growth and yield of *Triticum aestivum* var. Pusa Gold. J. Appl. Phycol..

[17] Kumari R., Kaur I., Bhatnagar A.K. (2011). Effect of aqueous extract of *Sargassum johnstonii* Setchell & Gardner on growth, yield and quality of *Lycopersicon esculentum* Mill. J. Appl. Phycol..

[18] Hernández-Herrera R.M., Santacruz-Ruvalcaba F., Ruiz-López M.A., Norrie J., Hernández-Carmona G. (2013). Effect of liquid seaweed extracts on growth of tomato seedlings (*Solanum lycopersicum* L.). J. Appl. Phycol..

[19] Barone V., Baglieri A., Stevanato P., Broccanello C., Bertoldo G., Bertaggia M., Cagnin M., Pizzeghello D., Moliterni V.M.C., Mandolino G. (2018). Root morphological and molecular responses induced by microalgae extracts in sugar beet (*Beta vulgaris* L.). J. Appl. Phycol..

[20] Barone V., Puglisi I., Fragalà F., Stevanato P., Baglieri A. (2019). Effect of living cells of microalgae or their extracts on soil enzyme activities. Arch. Agron. Soil Sci..

[21] Puglisi I., La Bella E., Rovetto E.I., Lo Piero A.R., Baglieri A. (2020). Biostimulant effect and biochemical response in lettuce seedlings treated with a *Scenedesmus quadricauda* extract. Plants.

[22] Santos P.L.F., Zabotto A.R., Jordão H.W.C., Boas R.L.V., Broetto F., Tavares A.R. (2019). Use of seaweed-based biostimulant (*Ascophyllum nodosum*) on ornamental sunflower seed germination and seedling growth. Ornam. Hortic..

[23] Parađiković N., Teklić T., Zeljković S., Lisjak M., Špoljarević (2019). M. Biostimulants research in some horticultural plant species—A review. Food Energy Secur..

[24] Lisjak M., Tomić O., Špoljarević M., Teklić T., Stanisavljević A., Balas J. (2015). Garden cress germinability and seedling vigour after treatment with plant extracts. Agriculture.

[25] Ronga D., Biazzi E., Parati K., Carminati D., Carminati E., Tava A. (2019). Microalgal biostimulants and biofertilisers in crop productions. Agronomy.

[26] Baglieri A., Cadili V., Monterumici C.M., Gennari M., Tabasso S., Montoneri E., Nardi S., Negre M. (2014). Fertilization of bean plants with tomato plants hydrolysates. Effect on biomass production, chlorophyll content and N assimilation. Sci. Hortic..

[27] Piccolo A., Conte P., Spaccini R., Mbagwu J.S.C. (2005). Influence of land use on the characteristics of humic substances in some tropical soils of Nigeria. Eur. J. Soil Sci..

[28] Islam A.K.M.M., Kato-Noguchi H. (2014). Phytotoxic activity of *Ocimum tenuiflorum* extracts on germination and seedling growth of different plant species. Sci. World J..

[29] Dürr C., Boiffin J., Fleury A., Coulomb I. (1992). Analysis of the variability of sugar beet (*Beta vulgaris*) growth during the early stages. Agronomy.

[30] Stibbe C., Märländer B. (2002). Field emergence dynamics significance to intraspecific competition and growth efficiency in sugar beet (*Beta vulgaris* L.). Euro. J. Agron..

[31] Romano A., Stevanato P. (2020). Germination Data Analysis by Time-to-Event Approaches. Plants.

[32] Podlaski S., Chomontowski C. (2020). Various methods of assessing sugar beet seed vigour and its impact on the germination process, field emergence and sugar yield. Sugar Tech..

[33] García A.C., de Souza L.G.A., Pereira M.G., Castro R.N., García-Mina J.M., Zonta E., Lisboa F.J.G., Berbara R.L.L. (2016). Structure-property-function relationship in humic substances to explain the biological activity in plants. Sci. Rep..

[34] Spaccini R., Piccolo A., Contea P., Haberhauer G., Gerzabek M.H. (2002). Increased soil organic carbon sequestration through hydrophobic protection by humic substances. Soil Biol. Biochem..

[35] Ugena L., Hýlová A., Podlešáková K., Humplík J.F., Doležal K., De Diego N., Spíchal L. (2018). Characterization of biostimulant mode of action using novel multi-trait High-Throughput screening of Arabidopsis germination and Rosette growth. Front. Plant Sci..

[36] Baglieri A., Sidella S., Barone V., Fragalà F., Silkina A., Nègre M., Gennari M. (2016). Cultivating *Chlorella vulgaris* and *Scenedesmus quadricauda* microalgae to degrade inorganic compounds and pesticides in water. Environ. Sci. Pollut. Res..

[37] Stanier R.Y., Kunisawa R., Mandel M., Cohen-Bazire G. (1971). Purification and properties of unicellular blue-green algae (order *Chroococcales*). Bacteriol. Rev..

[38] Barone V., Puglisi I., Fragalà F., Lo Piero A.R., Giuffrida F., Baglieri A. (2019). Novel bioprocess for the cultivation of microalgae in hydroponic growing system of tomato plants. J. Appl. Phycol..

[39] Puglisi I., Barone V., Sidella S., Coppa M., Broccanello C., Gennari M., Baglieri A. (2018). Biostimulant activity of humic-like substances from agro-industrial waste on *Chlorella vulgaris* and *Scenedesmus quadricauda*. Eur. J. Phycol..

[40] Hajizadeh H.S., Heidari B., Bertoldo G., Della Lucia M.C., Magro F., Broccanello C., Baglieri A., Puglisi I., Squartini A., Campagna G. (2019). Expression profiling of candidate genes in sugar beet leaves treated with Leonardite-based biostimulant. High-Throughput.

[41] The International Seed Testing Association (2020). ISTA International Rules for Seed Testing. ISTA 2020 Rules.

[42] Abbate C., Borzì D., Caboni P., Baglieri A., Gennari M. (2007). Behavior of fenhexamid in soil and water. J. Environ. Sci. Health B.

[43] Noorhosseini S.A., Jokar N.K., Damalas C.A. (2018). Improving seed germination and early growth of garden cress (*Lepidium sativum*) and Basil (*Ocimum basilicum*) with hydropriming. J. Plant Growth Regul..

[44] Maguire J.D. (1962). Speed of germination—Aid in selection and evaluation for seedling emergence and vigor. Crop Sci..

[45] Soltani E., Ghaderi-Far F., Baskin C.C., Baskin J.M. (2015). Problems with using mean germination time to calculate rate of seed germination. Aust. J. Bot..

[46] Ruan S., Xue Q., Tylkowska K. (2002). The influence of priming on germination of rice (*Oryza sativa* L.) seeds and seedling emergence and performance in flooded soil. Seed Sci. Technol..

[47] Chiapusio G., S´anchez A.M., Reigosa M.J., Gonzalez L., Pellissier F. (1997). Do germination indices adequately reflect allelochemical effects on the germination process?. J. Chem. Ecol..

[48] Coolbear P., Francis A., Grierson D. (1984). The effect of low temperature pre-sowing treatment on the germination performance and membrane integrity of artificially aged tomato seeds. J. Exp. Bot..

[49] Barone V., Bertoldo G., Magro F., Broccanello C., Puglisi I., Baglieri A., Cagnin M., Concheri G., Squartini A., Pizzeghello D. (2019). Molecular and morphological changes induced by Leonardite-based biostimulant in *Beta vulgaris* L.. Plants.

