# Supplementary material for: Effect of Microalgal Extracts from Chlorella vulgaris and Scenedesmus quadricauda on Germination of Beta vulgaris Seeds
Source: Plants (Basel). 2020 May 26;9(6):675. doi: 10.3390/plants9060675 (PMC7355607; doi:10.3390/plants9060675)
Supplement: Supplementary file 1 [file plants-09-00675-s001.pdf]

Supplementary Files

Supplementary Table 1. Distribution of C intensity of 13C NMR (%) of *Chlorella vulgaris* (CVextr) and *Scenedesmus quadricauda* (SQextr) extracts (Barone et al., 2018).

	Alkyl 0-45 ppm	N and O alkyl 45-95 ppm	Aromatic 95-160 ppm	Carboxyl 160-195 ppm	HB/HI[1]
**CVextr**	63.39	4.75	22.5	9.37	6.1
**SQextr**	55.23	16.25	24.09	4.42	3.8

[1] Hydrophobicity index was calculated as follows: HB/HI = [(0 – 45) + (95 – 160)/(45 – 95) + (160 – 195)].

Supplementary Table 2. Element composition (%) of *Chlorella vulgaris* (CVextr) and *Scenedesmus quadricauda* (SQextr) extracts (Barone et al., 2018).

	C	N	P	S	Mg	Ca	Fe	K	Na
**CVextr**	62.2	1.37	0.24	0.37	0.51	0.05	0.01	0.52	3.87
**SQextr**	62.8	1.17	0.36	0.41	0.53	0.15	0.01	0.35	2.42
